# Absence of left bundle branch block and blood urea nitrogen predict improvement in left ventricular ejection fraction in patients with cardiomyopathy and wearable cardioverter defibrillators

**DOI:** 10.1002/clc.23295

**Published:** 2019-12-20

**Authors:** Nikhil A. Mehta, Nashwa Abdulsalam, Ruth Kouides, Hamdy Ahmed, Raisa Atif, Abrar Shah, Sarah Taylor, Dmitry Chuprun, David Huang, Mohan Rao

**Affiliations:** ^1^ Department of Cardiology Lehigh Valley Health Network Allentown Pennsylvania; ^2^ Department of Cardiology University of Nebraska Medical Center Omaha Nebraska; ^3^ Department of Internal Medicine Rochester Regional Health Rochester New York; ^4^ Department of Electrophysiology Rochester Regional Health Rochester New York; ^5^ Department of Electrophysiology University of Rochester School of Medicine Rochester New York

**Keywords:** blood urea nitrogen, cardiomyopathy, left bundle branch block, ventricular arrhythmia, wearable Cardioverter defibrillator

## Abstract

**Objective:**

To identify predictors of left ventricular ejection fraction (LVEF) improvement in patients with newly detected cardiomyopathy using wearable cardioverter defibrillators (WCDs).

**Background:**

WCDs are useful in preventing sudden cardiac death in patients with reduced LVEF <35% while awaiting implantable cardioverter defibrillator (ICD) placement. In many patients, LVEF improves and an ICD is not indicated.

**Methods:**

Patients who received WCDs from November 2013 to November 2015 were identified and followed over a period of 2 years. Clinical variables were examined. The primary outcome was improvement in LVEF ≥35%. Predictors of outcome were determined using a multivariate logistic regression model.

**Results:**

A total of 179 patients were followed. Median age was 65 (interquartile range [IQR]: 56, 73) years, 69.3% were men. Median baseline LVEF was 20% (IQR: 15, 30). LVEF improved ≥35% in 47.5% patients, with patients being younger (62 vs 68.5 years, *P* = .006), having lower blood urea nitrogen (BUN) (19 vs 24 mg/dL, *P* = .002), fewer left bundle branch block (LBBB 9.5% vs 25.8%, *P* = .004), shorter QRS duration (98 vs 112 ms, *P* < .001), and higher use of angiotensin converting enzyme inhibitors (ACEI)/angiotensin receptor blockers (ARB) (92.9% vs 74.4%, *P* = .001) compared to those without LVEF improvement. Absence of LBBB (odds ratio [OR] 0.28, 95% confidence interval [CI] 0.11‐0.70), lower BUN (OR 0.13, 95% CI 0.02‐0.76), and ACEI/ARB use (OR 3.53, 95% CI 1.28‐9.69) were identified as independent predictors. Ventricular tachycardia/ventricular fibrillation was observed in three patients, all of whom received successful WCD shocks.

**Conclusion:**

Absence of LBBB, lower BUN, and ACEI/ARB use predicts LVEF improvement. WCDs help treat arrhythmic events.

## 1 INTRODUCTION

A left ventricular ejection fraction (LVEF) <35% is associated with increased risk of sudden cardiac death (SCD) in patients with newly diagnosed symptomatic heart failure.^1^, ^2^ As LVEF improves, the risk for heart failure hospitalization and SCD decreases.^1^ Current guidelines recommend implantable cardioverter defibrillator (ICD) placement for primary prevention of SCD in patients with symptomatic heart failure and LVEF <35%.^3^ In patients with ischemic cardiomyopathy, the DINAMIT and IRIS studies showed some arrhythmic benefit, but no mortality benefit with immediate ICD implantation.^4^, ^5^ Therefore, current guidelines recommend a waiting period prior to ICD implantation during which patients can still be at risk for SCD. It is during this waiting period that wearable cardioverter defibrillators (WCDs), or “lifevests,” are used as a bridge to ICD placement.^6^, ^7^ WCDs have been shown to successfully treat arrhythmic events from ventricular tachycardia (VT) or ventricular fibrillation (VF) in several observational studies including the WEARIT/BIROAD, aggregate U.S. nationwide data from the manufacturer (ZOLL, Pittsburgh, PA), and WEARIT II.^8^, ^9^, ^10^ However, the VEST trial, which was the first randomized clinical trial looking at ischemic cardiomyopathy patients with reduced LVEF wearing WCDs, did not show any benefit of the WCD reducing arrhythmic deaths (1.6% vs 2.4%, *P* = .18).^11^ Overall, strong clinical data on the effectiveness of WCDs is lacking.

The WEARIT II study also showed that nearly half of the patients with cardiomyopathy had improvement in LVEF, following which an ICD was no longer indicated.^10^ Optimal medical therapy using beta‐blockers (BB), angiotensin converting enzyme inhibitors (ACEI), angiotensin receptor blockers (ARB), angiotensin receptor‐neprilysin inhibitors (ARNI), aldosterone antagonists, and cardiac resynchronization therapy (CRT) has been shown to improve LVEF and overall survival in patients with moderate to severe left ventricular dysfunction.^12^ However, data is limited regarding the clinical variables that predict improvement in LVEF. A sub‐study of the MADIT CRT trial showed that systolic blood pressure (SBP) and left bundle branch block (LBBB) predicted improvement in LVEF in patients who received an ICD.^13^ Our study aimed to determine the clinical predictors of improvement in LVEF in patients with newly diagnosed cardiomyopathy who were prescribed a WCD. We further looked at the effectiveness of WCDs in preventing arrhythmic events in this patient population.

## 2 METHODS

### 2.1 Study population

We identified all patients above 18 years of age at Rochester Regional Health (RRH) Hospitals with newly diagnosed cardiomyopathy (LVEF <35%) who received WCDs (product of ZOLL Medical Corporation Inc.) for primary prevention of SCD between November 2013 and November 2015. Technical details of the WCD have been previously described.^7^ Patients who did not have follow up within the RRH network were excluded. The institutional review board approved the study, and a waiver of consent was obtained. All patients were expected to receive guideline‐directed medical treatment for reduced LVEF, and WCDs were prescribed for primary prevention of SCD. WCD placement was based on physician discretion and a shared doctor‐patient decision making process.

### 2.2 Data collection and follow up

Clinical variables, ECG, and echocardiographic data for all patients were collected from electronic health records. Patients were followed for a period of 2 years or until their LVEF recovered (>50%), whichever came sooner. Echocardiographic data was used to assess baseline LVEF at the time of WCD placement as well as subsequent LVEF measurements. Improvement in LVEF was defined as LVEF ≥35%, and an increase of ≥10% from their baseline LVEF. Final change in LVEF was determined by the difference between the patient's initial and subsequent LVEF measurements. WCD data that included arrhythmias (VT or VF), shock therapy, and nonshockable rhythms (pulseless electrical activity, asystole) were obtained from the RRH cardiac electrophysiology device management division. The clinical decision for ICD implantation was based on ACC/AHA/HRS guidelines for primary prevention of SCD in patients with cardiomyopathy, or secondary prevention of SCD in patients demonstrating sustained VT, VF, or appropriate WCD shocks.^14^

### 2.3 Statistical analysis

Minitab 16 (Minitab Inc.) was used for all data analysis. Categorical data was expressed as frequencies and percentages, while continuous data was expressed as median (and interquartile range) or mean (and SD), depending on the normality of distribution. A univariate analysis of baseline clinical characteristics was performed using Chi‐square or Fischer exact test for dichotomous variables, standard *t*‐test for normal continuous variables, and Kruskal‐Wallis for nonparametric variables. A two‐sided *P* < .05 was considered statistically significant. Univariate variables with a *P* < .1 were then entered into a multivariate logistic regression model, to determine independent predictors of LVEF improvement. All nonnormal continuous data was transformed using statistical functions to normalize the distribution. Results were expressed as odds ratios (with 95% confidence intervals) with respective *P* values. WCD data on the frequency of arrhythmias represents the percentage of patients who experienced an event, and the type of event.

## 3 RESULTS

Over the 2‐year period, 269 patients were identified to have new onset cardiomyopathy with LVEF <35% that were prescribed WCDs. One hundred seventy‐nine patients met the inclusion criteria and had complete data sets to be included in the statistical analysis. Of the 90 patients excluded, 13 were lost to follow up and 77 were limited by either incomplete data or follow up outside the RRH network.

Table 1 shows the baseline characteristics of the study population. Median age was 65 (interquartile range [IQR]: 56, 73) years, with 69.3% being male. Median LVEF at the time of WCD placement was 20% (IQR: 15, 30). Median follow up duration was 92 (IQR: 59, 151) days, and median follow up LVEF was 35% (IQR 20, 45). Ischemic cardiomyopathy (ICM) was found in 54.2%, and nonischemic cardiomyopathy (NICM) was found in 45.8%. Median blood urea nitrogen (BUN) was 20 (IQR: 16, 27.25) mg/dL and creatinine (Cr) was 1.0 (IQR: 0.8‐1.2) mg/dL. About 4.5% (8/179) patients had end‐stage renal disease (ESRD). LBBB was present in 18.1% (32/179) patients. The primary outcome of LVEF improvement was found in 47.5% (85/179) patients. In patients with LVEF improvement, 11.8% improved within 40 days, 38.8% improved in 40‐90 days, and 49.4% improved beyond 90 days. LVEF did not improve in 52.5% (94/179) patients.

**Table 1:** Baseline characteristics

	n = 179
Demographics	
Age median (IQR), year	65 (56, 73)
BMI median (IQR), kg/m^2^	29.36 (25, 33.7)
Male sex, % (n)	69.3 (124)
Clinical parameters, % (n)	
Diabetes mellitus	35.2 (63)
Hypertension	72.6 (130)
Hyperlipidemia	46.4 (83)
PVD	8.9 (16)
Atrial fibrillation	21.3 (38)
COPD	13.4 (24)
Anemia	18.9 (24)
CABG	16.8 (30)
PCI	40.2 (72)
Tobacco	71.2 (126)
ESRD	4.5 (8)
Laboratory data	
Creatinine median (IQR), mg/dL	1.0 (0.8, 1.2)
BUN median (IQR), mg/dL	20 (16, 27.25)
Sodium median (IQR), mEq/L	140 (138, 142)
Potassium median (IQR), mEq/L	4.15 (3.9, 4.4)
Ischemic xcardiomyopathy, % (n)	54.2 (97)
Ejection fraction (EF), % median (IQR)	20 (15, 30)
<20, % (n)	30.2 (54)
20‐24, % (n)	24.0 (43)
25‐25, % (n)	17.3 (31)
>30, % (n)	28.5 (51)
SBP median (IQR), mm Hg	118 (108, 130)
DBP median (IQR), mm Hg	70 (60, 80)
Follow up EF, % median (IQR)	35 (20, 45)
Duration b/w 2 echos	92 (59, 151)
EKG findings	
LBBB, % (n)	18.1 (32)
QRS median (IQR), ms	104 (92, 130)
QTc mean ± *SD*, ms	474.5 ± 36.7
Medications, % (n)	
Beta blockers	97.8 (175)
Carvedilol	66.9 (117)
Metoprolol	31.4 (55)
Digoxin	17.3 (31)
ACEI/ARB	83.2 (149)
Diuretics	69.3 (124)
Spironolactone	32.9 (59)
Hydralazine	5.6 (10)

Abbreviations: ACEI, angiotensin converting enzyme inhibitors; ARB, angiotensin receptor blockers; BUN, blood urea nitrogen; CABG, coronary artery bypass graft; COPD, chronic obstructive pulmonary disease; DBP, diastolic blood pressure; EKG, electrocardiography; ESRD, end‐stage renal disease; IQR, interquartile range; LBBB, left bundle branch block; PCI, percutaneous coronary intervention; PVD, peripheral vascular disease; SBP, systolic blood pressure.

In 97 patients with ICM, 41.2% (40 patients) showed LVEF improvement over a median duration of 84 days (IQR 45, 120). Of these, 17.5% improved within 40 days, 40% improved in 40‐90 days, and 42.5% improved beyond 90 days. Coronary artery revascularization occurred in 87.6%. In 82 patients with NICM, 54.9% (45 patients) showed LVEF improvement over a median duration of 104.5 days (IQR 64.5, 184.5). Of these, 44.4% improved within 90 days, 35.6% improved in 90 days‐9 months, and 20% improved beyond 9 months.

Table 2 shows the clinical characteristics of patients with and without LVEF improvement. Baseline LVEF was similar between the two groups (20% [IQR: 15, 30] vs 24% [IQR: 15, 30], *P* = .24), whereas LVEF at follow up was 45% (IQR: 40, 50) in the LVEF improvement group vs 20% (IQR: 20, 25) in the LVEF nonimprovement group (*P* < .001). Patients experiencing LVEF improvement were younger (median 62 years vs 68.5 years, *P* = .006), less likely to have anemia (7.0% vs 19.1%, *P* = .01), or chronic obstructive pulmonary disease (COPD, 11.8% vs 25.5%, *P* = .01). There was a trend towards those patients with ischemic cardiomyopathy being less likely to recover (47.1% vs 60.6%, *P* = .07). Patients with LVEF improvement had a similar SBP of 122 (IQR: 108, 137) vs 117 (IQR: 107.5, 127.5) mm Hg, *P* = .09, a higher diastolic blood pressure of 74 (IQR: 61, 81.5) vs 69 (IQR: 60, 77) mm Hg, *P* = .03, a lower BUN of 19 (IQR: 14.5, 26) vs 24 (IQR: 17.5, 33) mg/dL, *P* = .002, and a lower serum creatinine of 0.9 (IQR: 0.8, 1.2) vs 1.0 (IQR: 0.8, 1.3) mg/dL, *P* = .03 when compared with the LVEF nonimprovement group. Patients with LVEF improvement showed less LBBB (9.5% vs 25.8%, *P* = .004), shorter QRS (98 vs 112 ms, *P* < .001), and shorter QTc (464.8 vs 483.3 ms, *P* = .001) when compared to those without LVEF improvement. ACEI or ARB use was more prevalent in those with LVEF improvement (92.9% vs 74.4%, *P* = .001). Both groups were equally treated with BB (97.7% vs 97.9%, *P* = .94), with a greater percentage of patients with LVEF improvement being on metoprolol vs carvedilol (38.6% vs 25%, *P* = .05). In ICM patients, both groups had similar rates of coronary intervention.

**Table 2:** Left ventricular ejection fraction (LVEF) improvement vs no LVEF improvement

	EF improved (n = 85)	EF not improved (n = 94)	P value
Age median (IQR), years	62 (55.5, 69.5)	68.5 (58, 76)	.006*
BMI median (IQR), kg/m^2^	28.86 (24.85, 34)	29.8 (25.3, 33.16)	.9
Male sex, % (n)	69.4 (59)	69.2 (65)	.96
DM, % (n)	32.9 (28)	37.2 (35)	.55
HTN, % (n)	72.9 (62)	72.3 (68)	.93
HLD, % (n)	43.5 (37)	48.9 (46)	.47
PVD, % (n)	5.9 (5)	11.7 (11)	.19
A fib, % (n)	16.7 (14)	25.5 (24)	.15
COPD, % (n)	7.0 (6)	19.1 (18)	.01*
Anemia, % (n)	11.8 (10)	25.5 (24)	.01*
CABG, % (n)	12.9 (11)	20.2 (19)	.19
PCI, % (n)	36.5 (31)	43.6 (41)	.33
Tobacco, % (n)	64.7 (55)	77.2 (71)	.07
ESRD, % (n)	1.2 (1)	7.5 (7)	.06
Cr median (IQR), mg/dL	0.9 (0.8, 1.15)	1 (0.81, 1.25)	.03*
BUN median (IQR), mg/dL	19 (14.5, 26)	24 (17.5, 33)	.002*
Na median (IQR), mEq/L	140 (138, 142)	140 (138, 142)	.46
K median (IQR), mEq/L	4.1 (3.9, 4.4)	4.2 (3.9, 4.4)	.3
Ischemic cardiomyopathy, % (n)	47.1 (40)	60.6 (57)	.07
EF median (IQR)	20 (15, 30)	24 (15, 30)	.24
<20, % (n)	28.2 (24)	31.9 (30)	—
20‐24, % (n)	22.4 (19)	25.5 (24)	—
25‐25, % (n)	17.6 (15)	17.1 (16)	—
>30, % (n)	31.8 (27)	25.5 (24)	—
SBP median (IQR), mm Hg	122 (108, 137)	117 (107.5, 127.5)	.09
DBP median (IQR), mm Hg	74 (61, 81.5)	69 (60, 77)	.03*
Follow up EF median (IQR)	45 (40, 50)	20 (20, 25)	.0001*
Duration b/w 2 echos median (IQR)	89 (60.5, 164)	97.5 (54.5, 139.8)	.36
EKG findings			
LBBB, % (n)	9.5 (8)	25.8 (24)	0.004*
QRS median (IQR), ms	98 (90, 112)	112 (97, 138)	0.0001*
QTc mean ± *SD*, ms	464.8 ± 34.0	483.3 ± 36.9	0.001*
Medications			
Beta blockers	97.7 (83)	97.9 (92)	0.94
Carvedilol, % (n)	59.0 (49)	73.9 (68)	0.04*
Metoprolol, % (n)	38.6 (32)	25.0 (23)	0.05*
Digoxin, % (n)	16.5 (14)	18.1 (17)	0.77
ACEI/ARB, % (n)	92.9 (79)	74.4 (70)	0.001*
ASA, % (n)	80.0 (68)	79.8 (75)	0.97
Diuretics, % (n)	67.1 (57)	71.3 (67)	0.54
Spironolactone, % (n)	32.9 (28)	32.9 (31)	0.99
Hydralazine, % (n)	4.7 (4)	6.3 (6)	0.75

Abbreviations: ACEI, angiotensin converting enzyme inhibitors; ARB, angiotensin receptor blockers; ASA, aspirin; BUN, blood urea nitrogen; CABG, coronary artery bypass graft; COPD, chronic obstructive pulmonary disease; DBP, diastolic blood pressure; DM, diabetes mellitus; EF, ejection fraction; EKG, electrocardiography; ESRD, end‐stage renal disease; HLD, hyperlipidemia; HTN, hypertension; IQR, interquartile range; LBBB, left bundle branch block; PCI, percutaneous coronary intervention; PVD; SBP, systolic blood pressure. Bold values are indicates statistical significance

A best fit multivariate regression analysis identified three variables as being independently associated with LVEF improvement (Table 3). The absence of LBBB (odds ratio [OR] 0.28, 95% confidence interval [CI] 0.11‐0.70), lower BUN (OR 0.13, 95% CI 0.02‐0.76), and use of an ACEI/ARB (OR 3.53, 95% CI 1.28‐9.69) were independently associated with improvement in LVEF.

**Table 3:** Logistic regression model

	Z score	*P* value	OR	95% CI
Constant	1.27	.204		
LBBB	−2.74	.006	0.28	0.11‐0.70
ACEI/ARB	2.45	.014	3.53	1.28‐9.69
BUN	−2.26	.024	0.13	0.02‐0.76

Abbreviations: ACEI, angiotensin converting enzyme inhibitors; ARB, angiotensin receptor blockers; BUN, blood urea nitrogen; CI, confidence interval; LBBB, left bundle branch block; OR, odds ratio.

Table 4 summarizes patient WCD data. VT/VF was observed in three patients (1.7%), all of whom received successful WCD therapy. The first patient experienced three episodes of sustained VT, with appropriate shocks delivered for each episode, and subsequently underwent ICD implantation. The second patient had two episodes of sustained VT, with appropriate shocks delivered on both occasions, but then stopped wearing the WCD and died of presumed SCD. The third patient had VF that was appropriately shocked, but then developed asystole and died. All three patients were noted to have ICM. Overall, 79 patients (44.1%) underwent ICD implantation for primary prevention of SCD, of which 69 (87.3%) did not have improvement in LVEF and 10 (12.7%) did have recovery of LVEF.

**Table 4:** Wearable defibrillator data

	EF improved (n = 85)	EF not improved (n = 94)	Total (n = 179)
Incidence of treated VT/VF, n (%)	0.0 (0)	3.2 (3)	1.7 (3)
Successful appropriate shocks, n (%)	0.0 (0)	100 (3)	100 (3)
Nonshockable rhythms, n (%)	0.0 (0)	33.3 (1)	33.3 (1)
ICD implantations, n (%)	11.8 (10)	72.4 (69)	44.1 (79)

Abbreviations: CI, confidence interval; EF, ejection fraction; ICD, implantable cardioverter defibrillator; OR, odds ratio; VF, ventricular fibrillation; VT, ventricular tachycardia.

## 4 DISCUSSION

Our study had the following important findings for patients with newly diagnosed cardiomyopathy and LVEF <35% with prescribed WCDs: (a) nearly half experienced improvement in LVEF ≥35%; (b) baseline LVEF and etiology of cardiomyopathy did not affect LVEF improvement; (c) a greater percentage of patients demonstrated LVEF improvement when the waiting period was extended beyond 90 days; (d) simple baseline clinical and laboratory data can be used to predict LVEF improvement; and (e) WCD use as a bridge to ICD implantation helps protect against arrhythmic events in high risk patients.

Previous studies have demonstrated reduced LVEF as a predictor of SCD in patients with cardiomyopathy.^1^, ^2^ ICD implantation has been shown to reduce the long term risk of SCD in sub studies of the MADIT‐II, however data is limited in the short term setting (<90 days).^4^, ^15^, ^16^ WCDs have therefore been used during this period. Similar to prior studies, we were able to demonstrate close to 50% patients showing LVEF improvement ≥35%.^10^, ^17^, ^18^ Furthermore, similar to the WEAR‐IT II study, we were also able to demonstrate the etiology of cardiomyopathy, ischemic vs nonischemic, not influencing LVEF improvement.

A few studies have tried to identify clinical predictors of LVEF improvement and reverse remodeling, but differ from our study in methodology and have mixed results: (a) Binkley et al compared 53 patients with dilated cardiomyopathy having stage C congestive heart failure with LVEF improvement ≥40% to 59 frequency‐matched patients without LVEF improvement and showed shorter QRS, female gender, NICM, absence of diabetes mellitus, and higher SBP associated with LVEF improvement.^19^ (b) Wilcox et al looked at congestive heart failure patients with LVEF ≤35%, measuring improvement in LVEF ≥10% irrespective of their baseline, and showed female sex, no prior myocardial infarction (MI), NICM, and no digoxin use associated with LVEF improvement.^20^ (c) Brenyo et al studied patients from the MADIT‐CRT with LVEF <30% and QRS ≥130 ms and showed shorter QRS, higher SBP, low creatinine, and NICM as predictors of left ventricular reverse remodeling.^21^

In patients with newly diagnosed cardiomyopathy prescribed a WCD, our study is the first to demonstrate the absence of LBBB, lower BUN, and use of ACEI/ARB as independent predictors of LVEF improvement. Previous studies that associated LBBB and higher BUN with a reduced likelihood of left ventricular (LV) reverse remodeling centered around patients getting ICDs after a waiting period, much later in the disease process.^22^, ^23^, ^24^, ^25^ Elevated BUN is often viewed as a prognostic marker that reflects an impaired hemodynamic status proportionate to the reduction in cardiac output.^26^ The neuro‐hormonal changes that occur in patients with heart failure and cardiomyopathy are reflected more on BUN than creatinine (which closely mimics estimated glomerular filtration rate—eGFR).^27^ LBBB on the other hand results in electrical and mechanical dyssynchrony.^28^, ^29^ Findings from the MADIT‐CRT have shown LBBB preventing LV reverse remodeling, and absence of LBBB or correction of LBBB using a CRT device correcting this “dyssynchrony” with improvement in LV function.^30^, ^31^ Another sub‐study of the MADIT‐CRT also showed increasing heart failure events in patients with LBBB, as compared to those with RBBB or nonspecific intraventricular conduction delay.^13^ Although a QRS duration ≥150 ms has been shown to negatively predict LVEF improvement, our study lacked statistical power to support this finding. A trend was observed in patients with LVEF improvement having a narrower QRS. With GDMT, we found ACEI/ARB use to be predictive of LVEF improvement, which is consistent with results from prior large scale randomized trials such as the CONSENSUS, CHARM, and SOLVD.^32^, ^33^, ^34^

Improvement in LVEF ≥35% after a defined waiting period impacts decision making for ICD implantation for primary prevention of SCD. Current ACC/AHA/HRS guidelines recommend a waiting period of 40 days in nonrevascularized post‐MI patients, and 90 days in revascularized post MI patients and NICM patients.^35^ The median duration between initial and subsequent LVEF assessment in our study was 92 days. Interestingly, nearly half of the patients with improvement in cardiac function (49.4%) showed LVEF improvement beyond 90 days. This was true for a subgroup analysis for both ICM and NICM patients as well. In ICM patients with LVEF improvement, 57.5% improved at 90 days and 42.5% improved beyond 90 days, while in NICM patients, 44.4% improved at 90 days and 55.6% improved beyond 90 days. These findings support an extended waiting period on GDMT when monitoring for LVEF improvement. As LVEF improves, the need for an ICD for primary prevention of SCD decreases. A study by Duncker et al also showed similar results, with approximately 1/3rd of the patients who received ICDs experiencing LVEF improvement beyond the currently recommended 90 day waiting period.^36^

During the waiting period, in patients with new onset cardiomyopathy, previous studies have found WCDs to be effective in treating ventricular arrhythmias.^8^, ^9^, ^10^ Three patients in our study (1.7%) experienced one or several arrhythmic episodes of VT/VF, with appropriate successful WCD shocks on all occasions. All three patients were noted to have ischemic cardiomyopathy. The aggregate US nationwide data and WEARIT‐II studies both reported a similar VT/VF event rate of 1.7%‐2.1%, especially in ICM and congenital heart disease patients as compared to other cardiomyopathy subgroups.^9^, ^10^ Patient compliance however is a limiting factor in their use. As noted in our study, one patient died from noncompliance. In current ACC/AHA/HRS guidelines, the use of a WCD in patients with new onset cardiomyopathy is a class IIb recommendation. We hope that our study offers supportive data for the use of WCDs to treat ventricular arrhythmias during the waiting period prior to ICD implantation.

Our study has the following limitations: (a) given its retrospective nature, we were unable to determine a sequential cause‐effect relationship; (b) our patient population was limited to a single center community healthcare establishment, with a percentage of patients lost to follow up outside the healthcare network; (c) the time to follow up LVEF measurement had some variance despite attempts to adhere to the guideline recommended 40 or 90 days that could not be accounted for in the analysis; and (d) despite several baseline clinical differences between patients with and without LVEF improvement, statistical power was limited by sample size in identifying other possible independent predictors on logistic regression analysis.

## 5 CONCLUSION

In conclusion, in patients with new onset cardiomyopathy, close to half the patients will experience improvement in LVEF. The time to LVEF recovery can extend well beyond 90 days. The absence of LBBB, lower BUN, and use of ACEI/ARB were found to be independent predictors of LVEF improvement. The WCD was effective in treating ventricular arrhythmias during the waiting period prior to ICD implantation.

## CONFLICT OF INTEREST

The authors declare no potential conflict of interests.
